# Protective Effects of Enriched Environment Against Transient Cerebral Ischemia-Induced Impairment of Passive Avoidance Memory and Long-Term Potentiation in Rats

**DOI:** 10.29252/nirp.bcn.8.6.443

**Published:** 2017

**Authors:** Ali Ahmadalipour, Jafar Sadeghzadeh, Seyed Afshin Samaei, Ali Rashidy-Pour

**Affiliations:** 1. Research Center of Psychiatry and Behavioral Sciences, Tabriz University of Medical Sciences, Tabriz, Iran.; 2. Students Research Committee, Semnan University of Medical Sciences, Semnan, Iran.; 3. Department of Neurology, School of Medicine, Semnan University of Medical Sciences, Semnan, Iran.; 4. Neuromuscular Rehabilitation Research Center, Semnan University of Medical Sciences, Semnan, Iran.; 5. Laboratory of Learning and Memory, Physiology Research Center, School of Medicine, Semnan University of Medical Sciences, Semnan, Iran.

**Keywords:** Enriched environment, Brain ischemia, Long-term potentiation, Memory

## Abstract

**Introduction:**

Enriched Environment (EE), a complex novel environment, has been demonstrated to improve synaptic plasticity in both injured and intact animals. The present study investigated the capacity of an early environmental intervention to normalize the impairment of passive avoidance memory and Long-Term Potentiation (LTP) induced by transient bilateral common carotid artery occlusion (2-vessel occlusion, 2VO) in rats.

**Methods:**

After weaning, young Wistar rats (22 days old) were housed in EE or Standard Environment (SE) for 40 days. Transient (30-min) incomplete forebrain ischemia was induced 4 days before the passive avoidance memory test and LTP induction.

**Results:**

The transient forebrain ischemia led to impairment of passive avoidance memory and LTP induction in the Perforant Path-Dentate Gyrus (PP-DG) synapses. Interestingly, housing and growing in EE prior to 2VO was found to significantly reverse 2VO-induced cognitive and LTP impairments.

**Conclusion:**

Our results suggest that early housing and growing in EE exhibits therapeutic potential to normalize cognitive and LTP abnormalities induced by 2VO ischemic model in rats.

## 1. Introduction

Ischemic stroke is one of the main causes of long lasting neurological defects, emotional, and memory dysfunction (Luo et al., 2007), as well as apathy (Mikami, Jorge, Moser, Jang, & Robinson, 2013), dizziness (Johkura et al., 2012) randomized, open-label, blinded endpoint trial. One hundred six patients who suffered supratentorial ischemic stroke within the previous 1–6 months and subsequently complained of persistent dizziness without other obvious sequelae were enrolled. Patients were randomly given cilostazol (200 mg/day), and headache (Ahmadi Aghangar, Bazoyar, Mortazavi, & Jalali, 2015) in humans. These impairments are presumably caused by neuronal and or functional damages; consequences of cerebral infarction. Learning and memory dysfunction affect the quality of life in the majority of patients with cerebral infarction, and memory deficits are often related to impaired hippocampal function (Snyder et al., 2015). In animal models, bilateral common carotid artery occlusion results in impairment of working memory in the adult rat (Sarti, Pantoni, Bartolini, & Inzitari, 2002; Yan et al., 2007).

Hippocampus, a brain structure, has an essential function in the brain network and is important for memory function. In addition, it is very sensitive to hypoxicischemic episodes (Erfani et al., 2015; Farkas, Luiten, & Bari, 2007; Sarti et al., 2002). Hippocampal neurons are not damaged instantly after transient ischemia, but neural death occurs in the next few days (Kirino, 1982). Neurobiological mechanisms underlying the impaired hippocampal function are poorly understood, but are important in terms of clinical respect to the development of effective rehabilitation of stroke patients. One mechanism possibly contributing to learning and memory deficits is impairment in the hippocampal Long-Term Potentiation (LTP). According to evidence, 4 days after transient ischemia, LTP was inhibited in the Perforant Path-Dentate Gyrus (PP-DG) and Schaffer collateral CA1 synapses (Mori, 1998).

LTP has been extensively thought to be responsible for the cellular mechanism of learning and memory. This phenomenon was first noted in the PP-DG synapses of anesthetized rabbits (Bliss & Lømo, 1973). The population response was analysed in terms of three parameters: the amplitude of the population Excitatory Post-Synaptic Potential (EPSP). As the PP is one of the main extrinsic inputs to hippocampal formation, an area of the brain thought to be involved with learning and memory; LTP has been speculated to be a principle component of memory (Bliss & Collingridge, 1993). LTP is facilitated by exposure to Enriched Environment (EE) or exercise (Yang et al., 2007).

Hebb was the first who reported the positive effects of EE in his paper in 1947 that the animals kept as pets in EE displayed improved performance in memory and learning tests (Hebb, 1947). Since then, hundreds of experimental data have accumulated regarding environmental factors and their significance. Among others, EE has been shown to impact the development of the nervous system (Ortuzar, Argando, Bengoetxea, & Lafuente, 2011). Moreover, environmental factors have a major influence on the outcome of different neuronal lesions (van Praag, Kempermann, & Gage, 2000). Recently, we have shown that postnatal EE improves memory impairment induced by prenatal exposure to morphine (Ahmadalipour et al., 2015) in a similar manner to the effects of postnatal treadmill exercise (Ahmadalipour & Rashidy-Pour, 2015).

Previous studies have also reported that EE produces morphological and functional alterations in the DG of young and adult animals. For instance, electrophysiological studies showed that exposure to EE regulates synaptic transmission, excitability, and LTP in the rat DG (Irvine, Logan, Ecket, & Abraham, 2006), but the physiological changes in neural function that may mediate these effects are poorly understood. To date, there have been conflicting reports regarding potential mechanisms, such as an increase in basal synaptic transmission, an increase in cell excitability, or altered synaptic plasticity. Here, we reexamined in freely moving animals the conditions under which varying degrees of EE exposure might lead to increases in synaptic or neural function in the dentate gyrus of the hippocampus.

Adult male Sprague-Dawley rats were chronically implanted with stimulating and recording electrodes in the perforant path and dentate gyrus, respectively, and housed singly in standard cages. After stable recordings were established for Field Excitatory Post-Synaptic Potentials (FEPSPs). Alterations in DG cells morphology can be induced by EE in the weaning and post-weaning period (Bartesaghi, Raffi, & Ciani, 2006; Bartesaghi & Severi, 2004), although data about adult animals are contradictory (Fiala, Joyce, & Greenough, 1978; Speisman et al., 2013).

Furthermore, involvement in the complex environment after ischemic injuries improves neural plasticity, including greater neurogenesis, reactive synaptogenesis and dendritic restructuring (Matsumori et al., 2006). However, it is still unclear whether adolescent exposure to EE can be used as an efficient preventive strategy against LTP function impairment associated with global hypoperfusion. Thus, the present study aimed to evaluate the protective effects of EE in adolescent period against the impairment of passive avoidance memory and LTP induced by transient cerebral ischemia in adult male rats.

## 2. Methods

### 2.1. Animals and housing conditions

**Figure 1.**
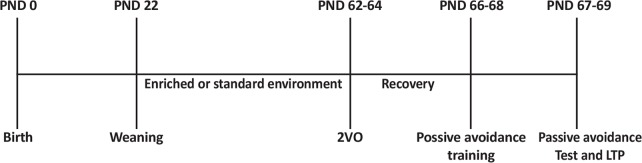
Timeline of the experimental design. After weaning at Postnatal Day (PND) 22, rats housed in Enriched Environment (EE) or Standard Environment (SE). The 2-Vessel Occlusion (2VO) was prepared at PNDs 62–64. Passive avoidance testing was performed at PNDs 66–68, which was followed by electrophysiological recordings at PNDs 67–68.

Male Wistar (n=30) rats were weaned at the age of 22 days and housed in EE or Standard Environment (SE) (Figure 1). The EE group was located in a large polycarbonate cage (100 cm×100 cm×50 cm) (12 rats in each cage) containing a running wheel, a raised platform, a group of plastic tunnels, steel chains, dissimilar size plastic balls and dolls changed every 5–6 days. The SE group was housed in normal Plexiglas cages (60 cm×40 cm×20 cm) (four rats in each cage). Rats were provided with water and food ad libitum, and kept in an automatic temperature-regulated room under a dark/light round (lights on/off at 6:00 AM/6:00 PM). Rats were dispersed into 3 experimental groups: 1. The SHAM/SE group: those animals which raised in SE and had the bilateral common carotid arteries exposed but received no additional manipulation; 2. The STR/SE group: those animals which raised in SE and had the bilateral common carotid arteries exposed and induced transient 2VO occlusion for 30 min; and 3. The STR/EE group: those animals which raised in EE and had transient 2 Vessel Occlusion (2VO) for 30 min.

### 2.2. Transient cerebral ischemia

A transient 2VO ischemic was induced at the age of 62–64 days by bilaterally clamping and blocking the common carotid arteries similar to the method of Mori et al. (Mori et al., 1998). The rats were anesthetized with chloral-hydrate (400 mg/kg, ip) and the bilateral common carotid arteries were exposed and clamped with microarterial surgical clips. Blood flow in the common carotid artery was reperfused by removing the clips after the 30 min occlusion. Rectal temperature was kept at around 37°C during this operation using a heating pad. Control rats in the sham operation group had the bilateral common carotid arteries exposed but no further manipulation was done.

### 2.3. Passive avoidance training and testing

Passive avoidance training was carried out 4 days (recovery period) after clamping the bilateral common carotid arteries. The experimental device consisted of a shuttle box^1^ divided into dark and light compartments. Both compartments had a grid floor (2-mm stainless steel rods spaced at 6-mm distance from each other) linked to an electrical shock producer. The apparatus was housed in a sound-attenuated room. Passive avoidance test consisted of a training trial on the first day, Post-Natal Day (PND) 66–68, and a retention test 24 h later. Before training, the rats were permitted to discover the apparatus without electric shock for 60 s.

During the training, each rat was placed in the light section of the apparatus facing opposite side of the door, and 5 s later, the sliding door was opened and the rat was allowed to cross-over into the dark section. After entering the dark section with all four paws, the sliding door was closed and the rat was given a foot shock (constant current, 1 mA for 2 s). Twenty seconds after shock, the rat was located in the cage and again placed inside of the light section 120 s later. If the rat did not enter the dark chamber during 120 s, the training trial was finished. Rats were tested for memory retention 24 h after training (PND 67–69). In the test session, the animals were placed in the light (safe) section, and the latency of entering the dark chamber (Step-Through Latency, STL) and the total Time spent in the Dark Compartment (TDC) were recorded. The ceiling score was 300 s.

### 2.4. Electrophysiology experiment

The electrophysiological experiments were carried out 2 days following the passive avoidance test (PNDs 67–69). For electrophysiological recording, stimulating and recording electrodes were prepared by gluing together a couple of twisted Teflon-coated 90% platinum and 10% iridium wires (135 μm). The animals were anesthetized with urethane (1–1.2 g/kg, ip) and stimulating electrode was implanted in the medial PP coordinates: Anteroposterior (AP), 7 mm; Mediolateral (ML), 4 mm; Dorsoventral (DV), 3–3.3 mm, from skull surface) and a recording electrode was implanted in the DG granule cell layer (coordinates: AP, 3; ML, 2; DV, 2.7–3.2 from skull surface) (Miladi Gorji, Rashidy Pour, Fathollahi, Semnanian, & Jadidi, 2014). In order to reduce trauma to brain tissue, we lowered the electrodes very gradually (0.2 mm/min). The correct placement of the electrodes was determined by physiological and stereotaxic indicators.

Relocation of the recording and or stimulating electrodes was done to obtain the highest potential and minimum variation in the Population Spike (PS) amplitude. The stimulus intensity that evoked a PS or Excitatory Post-Synaptic Potentials (EPSP) about 50% to 60% of the baseline maximal response was adjusted for the following training stimuli. Following electrode settlement (stabilization period), continuous current rectangular stimulus pulses were delivered for 20 min.

Extracellular field potentials were amplified, filtered (bandpass: 1 Hz-3 kHz), and sampled at a rate of 20 kHz and saved on the hard disk. All stimulation and recording were done using an on-line electronic oscilloscope-stimulator and data analysis interface system by NeuroTrace Software and Data Acquisition D3111 set up^2^. Next, the baseline synaptic responses had been constant for at least 20 min, High-Frequency Stimulation (HFS) was used for LTP induction consisted of 8 PBs at intervals of 10 s. Tetanus HFS consisted of 10 trains at 1 Hz composed each of 8 pulses at 400 Hz.

Tetanic stimulation is an effective protocol for inducing robust and persistent LTP, which is based on the physiology of hippocampus. After tetanus delivery, responses were recorded in PP-DG synapses, 5, 15, 30, 45, 60, 75, and 90 min after the HFS. The magnitude of potentiation was evaluated as the percentage change in the PS amplitude and the slope of EPSP relative to the pre-tetanus test value.

### 2.5. Statistical analysis

For analysis of the passive avoidance data, 1-way ANOVA with the Tukey post hoc test was used. For LTP measurements, EPSP slope and PS amplitude were expressed as a percentage of the 20-min baseline value before HFS application. Because a normal distribution for the electrophysiological data could not be assumed, a non-parametric test on two independent samples was selected (Mann Whitney U-test). A P-value of <0.05 was considered significant and SPSS 23.0 for Windows was used to perform all statistical analyses.

## 3. Results

### 3.1. Passive avoidance training and testing

**Figure 2.**
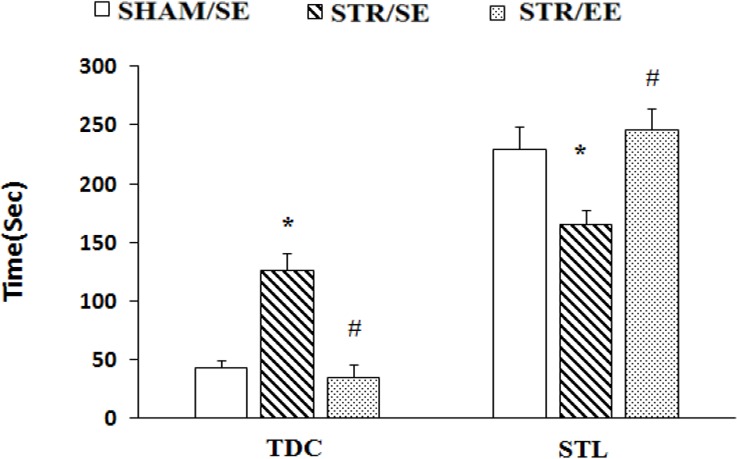
Protecting power of housing in Enriched Environment (EE) to prevent the impairment of avoidance memory in adulthood caused by global ischemia *P<0.05 the STR/SE group vs. the SHAM/SE group and #P<0.05 the STR/EE group vs. the STR/SE group; Data points are means±SEM of latency of entering the dark chamber (step-through latency, STL) and the total time spent in the dark compartment (TDC).

Data for passive avoidance memory of the experimental groups are shown in Figure 2. One-way ANOVA for the STL revealed significant differences between groups (F_2,24_=6.093, P=0.012). Furthermore, post hoc analysis revealed that transient cerebral ischemia significantly decreased STL in the STR/SE group than the SHAM/SE group (P=0.03), indicating that transient cerebral ischemia impaired passive avoidance memory. In addition, growing and housing in EE increased STL in the STR/EE group than the STR/SE group (P=0.026).

Similar results were found with TDC analysis. One-way ANOVA for the TDC showed significant differences between groups (F_2,24_=5.078, P=0.023). Furthermore, post hoc analysis revealed that transient cerebral ischemia significantly increased TDC in the STR/SE group compared with SHAM/SE group (P=0.03), indicating that transient cerebral ischemia impaired passive avoidance memory. In addition, growing and housing in EE increased TDC in the STR/EE group compared to STR/SE group (P=0.025).

### 3.2. In vitro electrophysiological recordings

#### 3.2.1 I/O curve

**Figure 3.**
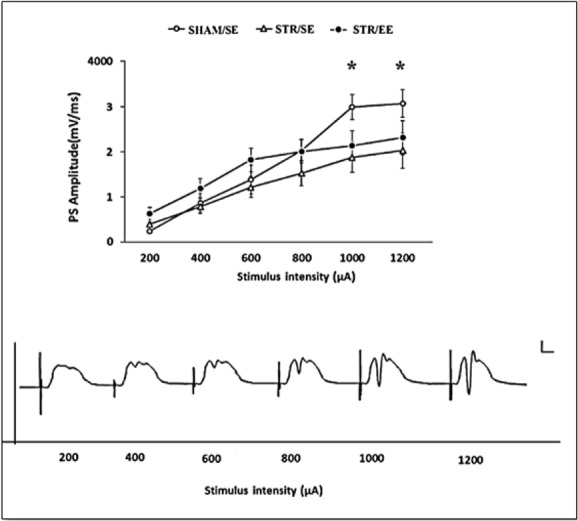
Input-output curves for Population Spike (PS) amplitudes B, an example of field potential traces (here Sham group) used to derive input-output curves in A. In A: *P<0.05 the SHAM/SE group vs. the STR/SE group. Scale bars: 2 mV/2 ms.

The PS amplitude is plotted as a function of stimulus pulse duration (Figure 3). Transient (30-min) incomplete forebrain ischemia affected I/O curves in the DG area measured by the PS amplitude. There were significant differences in stimulus-response curves in the DG measured as PS amplitude between the STR/SE group and the SHAM/SE group in high intensities (1000 and 1200 μA) (P<0.05). There was no significant difference in stimulus-response curves in the DG measured as EPSP slopes (Data are not shown).

#### 3.2.2 Alterations of the PS amplitude

**Figure 4.**
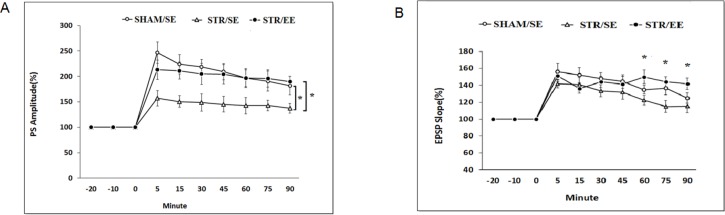
Housing in Enriched Environment (EE) in adolescent period prevent the Long-Term Potentiation (LTP) impairment of Population Spike (PS) (A) and EPSP (B) in adulthood caused by global ischemia In the control group, LTP was induced by High-Frequency Stimulation (HFS) of the perforant pathway (10 trains at 1 Hz composed each of 8 pulses at 400 Hz). The stroke (STR) group received only the 30-min 2-vessel occlusion 4 days earlier. The EE group, housed in EE for 40 days before 2-vessel occlusion. In A: *P<0.05 the SHAM/SE group than the STR/SE group in time points of 5 to 45 min, and the STR/SE group than the STR/EE group in most time points except time points 5 and 60 min that have a tendency to be significant (P=0.07). In B: *P<0.05 the STR/SE group than the STR/EE group in the late minutes (60–90 minutes). Data points are mean±SEM of normalized amplitude of PS or EPSPs.

In the STR/SE group, tetanus HFS stimulation resulted in a 157%±15 % increase in the PS amplitude, which gradually decreased reached to 138% of baseline at the end of the recording period. The same conditioning protocol resulted in a robust increase (250%±20%) in the PS amplitude in the SHAM/SE group (Figure 4A) which gradually decreased and reached to 180%±20% at the end of the recording period. A robust increase (213%±18 %) was also induced after HFS in the STR/EE which remained almost at elevated level throughout the 1.5-h recording period and reached to 190%±10% at the end. Between group comparisons using post hoc analysis revealed a significant decrease in the PS amplitude of the SHAM/SE group than the STR/SE group in time points of 5 to 45 minutes (P=0.05) and the STR/SE group compared to STR/EE group in most time points (P=0.05) except that the PS amplitude in time points of 5 and 60 minutes have a tendency to be significant (P=0.07).

#### 3.2.3 Alterations of the slope of EPSP

Tetanus HFS had no significant effect on EPSP slope recorded from the DG area of hippocampus in the STR/ST group compared to sham control rats (P>0.05), but there were significant differences in the STR/EE rats compared to STR/SE rats in 60, 75, and 90 min (P<0.05) (Figure 4B).

## 4. Discussion

Transient bilateral occlusion of the common carotid arteries in rats is a proven procedure to investigate the effects of temporary cerebral hypoperfusion on cognitive dysfunction and neurodegenerative processes. This study revealed that housing and growing in EE offers a protection against ischemic induced impairment of passive avoidance memory and LTP in rats.

The 2VO model described by Smith is a common technique for creation of transient ischemia model (Smith et al., 1984). However, this kind of occlusion is incomplete for rats compared with 4-vessel occlusion described by Pulsinelli and Brierly (Pulsinelli & Brierley, 1979) and it is reported that histological changes after the bilateral common carotid arteries clamping for 10 min were not distinguished using light microscopy (Mori et al., 1998).

The hippocampus is one of the brain parts most vulnerable to ischemic insults (Pulsinelli, Brierley, & Plum, 1982) and evidently four days after the 10 min clamping of the bilateral carotid arteries, the LTP reduced in both the PP-DG and the Schaffer collateral-CA1 synapses without any histological damages (Mori et al., 1998).

Our results demonstrated that transient ischemic stroke impaired stimulus-response (I/O) curves in high intensities in the DG of hippocampus (in the STR/SE compared with the SHAM/SE group). We also found that LTP induction measured by the PS amplitude in the stroke group was impaired as compared with the sham group.

Furthermore, 30 minutes ischemia caused an instant decrease in the levels of tyrosine phosphorylation and protein amount of both subunits NR2A and NR2B of NMDA receptor (NMDAr) (Zalewska, Ziemka-Nałȩcz, & Domańska-Janik, 2005). The tyrosine phosphorylation of NR2 subunits is a key component to govern the activity of NMDAr channel and gates the construction of NMDAr-dependent synaptic plasticity and potentiation (Kalia & Salter, 2003).

According to previous studies, brain parts which support memory are exclusively sensitive to oxidative stress because of their higher oxygen demands (Urso & Clarkson, 2003; Vannucci & Vannucci, 1997). The hippocampus is a brain structure specifically vulnerable to ischemia-induced oxidative stress. Behavioral studies in animals have confirmed that hippocampal damage results in impairment of learning and memory (Greenamyre, Olson, Penney, & Young, 1985), particularly on tasks that involve place learning (Yoo et al., 2010). For instance, Sarkaki et al. demonstrated that initial latency (learning) and step-through latency (memory) impaired after permanent bilateral common carotid arteries occlusion in adult female rats (Sarkaki, Rezaiei, Gharibnaseri, & Rafieirad, 2013).

Two-vessel occlusion has been also found to increase NMDA receptor density in the hippocampus (Farkas, Luiten, & Bari, 2007) which play an important physiological role in memory (Collingridge, Kehl, & McLennan, 1983). Additionally, glutamate release in the brain tissue increases following cerebral ischemia (Davalos, Shuaib, & Wahlgren, 2000). This ischemia-induced release of glutamate perhaps occurs in human as well (Chun, Kim, Choi, & Chang, 2008), and perhaps underlies selective impairment to the memory and hippocampal function.

Since brain development is reactive to environmental stimuli (Meaney & Aitken, 1985), the probability that environmental stimulation would act against the impairment of learning and memory and LTP by transient cerebral ischemia has been pursued. EE, as a motivation pattern, includes a combination of increased social interaction, long lasting contact to learning tasks, and physical exercise that produces interesting effects (Rojas et al., 2013).

Numerous investigations have studied the advantageous and neuroprotective effects of EE along with animal models of various insults, such as early-life stress (Cui et al., 2006), prenatal exposure to opioids (Ahmadalipour et al., 2015) or in different brain injury models, including stroke (Matsumori et al., 2006), epilepsy (Wang et al., 2007), Alzheimer disease (Beauquis et al., 2013), Parkinson disease (Faherty, Shepherd, Herasimtschuk, & Smeyne, 2005), Huntington disease (Hockly et al., 2002), and so on. Enhanced learning and memory caused by EE is one of the most consistent findings in the literature (van Praag et al., 2000). It would seem that EE reverses the detrimental action of early inconsistent stimulation and increases the advantageous effects of postnatal handling on shuttle box learning in adult rats (Escorihuela, Tobeña, & Fernández-Teruel, 1994). EE experience improves learning deficits and depressive-like behavior induced by juvenile stress (Ilin & Richter Levin, 2009). Involvement in the complicated environment following ischemic injuries improved neural plasticity, including increased neurogenesis, dendritic restructuring and reactive synaptogenesis (Matsumori et al., 2006).

In our study, the exposure to EE prevented the impairment of avoidance memory and hippocampal LTP associated with global hypoperfusion. EE can likely induce its protective effect through different ways at the same time. These mechanisms may involve the molecular changes, such as augmented number of Fos-positive neurons in the DG (Puurunen, Koistinaho, Sirviö, Jolkkonen, & Sivenius, 2001), upregulation of a candidate-plasticity genes such as early gene arc in the hippocampus (Pinaud, Penner, Robertson, & Currie, 2001), and overexpression of the Vascular Endothelial Growth Factor (VEGF) in hippocampal area, which acts as a neurotrophic factor (Cao et al., 2004).

It seems that EE upregulates hippocampal LTP (Duffy, Craddock, Abel, & Nguyen, 2001), suggesting a role for EE in regulating synaptic plasticity. Also, EE has been shown to increase the Brain Derived Neurotrophic Factor (BDNF), which increases mRNA expression and protein levels of NMDAr subunits, NR1, NR2A and NR2B (Caldeira et al., 2007). By increasing the number of NMDAr, EE may compensate low levels of protein and tyrosine phosphorylation of NR2A subunit of NMDAr and return to normal the impaired LTP in DG induced in the 2VO.

The brain tissue has been demonstrated to be sensitive to oxidative stress and several studies have shown that oxidative damage plays an important role in the pathogenesis of many neurodegenerative diseases such as stroke, vascular dementia, and Alzheimer disease (Chung et al., 2005; Coyle & Puttfarcken, 2014). Oxidative stress is defined as the imbalance between oxidants and antioxidants in favor of oxidant activity that potentially results in tissue damage (Polidori, Mecocci, Cherubini, & Senin, 2000). Interestingly, EE has been shown to prevent behavioral deficits and oxidative stress caused by Chronic Cerebral Hypoperfusion (CCH) in rats (Fernández, Collazo, Bauza, Castellanos, & López, 2004). Superoxide Dismutase (SOD) is one of the key factors involved in the antioxidant system and is critical for the protection of the brain tissue from oxidative damage. It has been demonstrated that EE can affect the regulation of SOD activity in rats submitted to CCH (Cechetti et al., 2012).

Another mechanism which possibly underlies the protective effect of EE is the increase of the levels of BDNF. In addition to its well-documented neuroprotective action, several experimental data indicate a role for BDNF in activity-dependent processes (Lu, 2003), such as synaptic plasticity (Karpova, 2014). Persumably, BDNF facilitates LTP and cognitive functions after transient forebrain ischemia (Kiprianova, 1999). It is demonstrated that CCH resulted in decreased levels of BDNF and NMDA receptor subunit 1 (NR1) protein in the hippocampus, and EE exposure restored the decreased expression of these molecules (Sun et al., 2010). Therefore, BDNF and NR1 may contribute to the beneficial effects of EE on CCH in rats.

In this study, the short transient brain hypoperfusion resulted in impairment of avoidance memory and reduced PS amplitude as an impaired LTP function in the PP-DG synapses. Interestingly, housing and growing in EE during adolescent period prior to 2VO protected the impairment of avoidance memory and restored the impaired LTP nearly to the control level. Our results suggest that early housing and growing in EE exhibits therapeutic potential to normalize the impaired memory and LTP in DG induced in the 2VO ischemic model in rats. To clarify the exact mechanism of action of EE and 2VO ischemic on the learning and memory and LTP induction, further experiments are needed.

## Footnotes

1. http://www.Iranlabexpo.ir

2. http://www.ScienceBeam.com
